# 3-Methyl-4-(2-phenyl-1,2,4-triazolo[1,5-*a*]pyrimidin-7-yl)furazan

**DOI:** 10.1107/S1600536813027700

**Published:** 2013-10-16

**Authors:** Kyrill Yu. Suponitsky, Victor M. Chernyshev, Nadezhda V. Palysaeva, Aleksei B. Sheremetev

**Affiliations:** a A. N. Nesmeyanov Institute of Organoelement Compounds, Russian Academy of Sciences, 28 Vavilova St, 119991 Moscow, Russian Federation; b South-Russia State Technical University, 346428 Novocherkassk, Russian Federation; c N. D. Zelinsky Institute of Organic Chemistry, Russian Academy of Sciences, 47 Leninsky Prosp., 119991 Moscow, Russian Federation

## Abstract

In the title mol­ecule, C_14_H_10_N_6_O, the planes of the methyl­furazan fragment and the phenyl ring attached to the triazolo­pyrimidine bicycle are twisted from the mean plane of the bicycle at angles of 45.92 (5) and 5.45 (4)°, respectively. In the crystal, π–π inter­actions, indicated by short distances [in the range 3.456 (3)–3.591 (3) Å] between the centroids of the five- and six-membered rings of neighbouring mol­ecules, link the mol­ecules into stacks propagating along the *c-*axis direction.

## Related literature

For applications of enamino­nes in synthesis, see: Kulinich & Ischenko (2009 ▶); Stanovnik & Svete (2004 ▶). For the synthesis of triazolo­pyrimidines from enamino­propenones, see: Abdelhamid *et al.* (2012 ▶, 2013 ▶); Behbehani & Ibrahim (2012 ▶). For X-ray studies of [1,2,4]triazolo[*a*]pyrimidines, see: Lipkind *et al.* (2011 ▶); Shikhaliev *et al.* (2008 ▶); Lokaj *et al.* (2006 ▶) and of furazan derivatives, see: Sheremetev *et al.* (2004 ▶, 2006 ▶, 2012 ▶, 2013 ▶); Suponitsky *et al.* (2009*a* ▶,*b* ▶). For normal values of bond lengths in organic compounds, see: Allen *et al.* (1987 ▶) and for a description of the Cambridge Structural Database, see: Allen (2002 ▶).
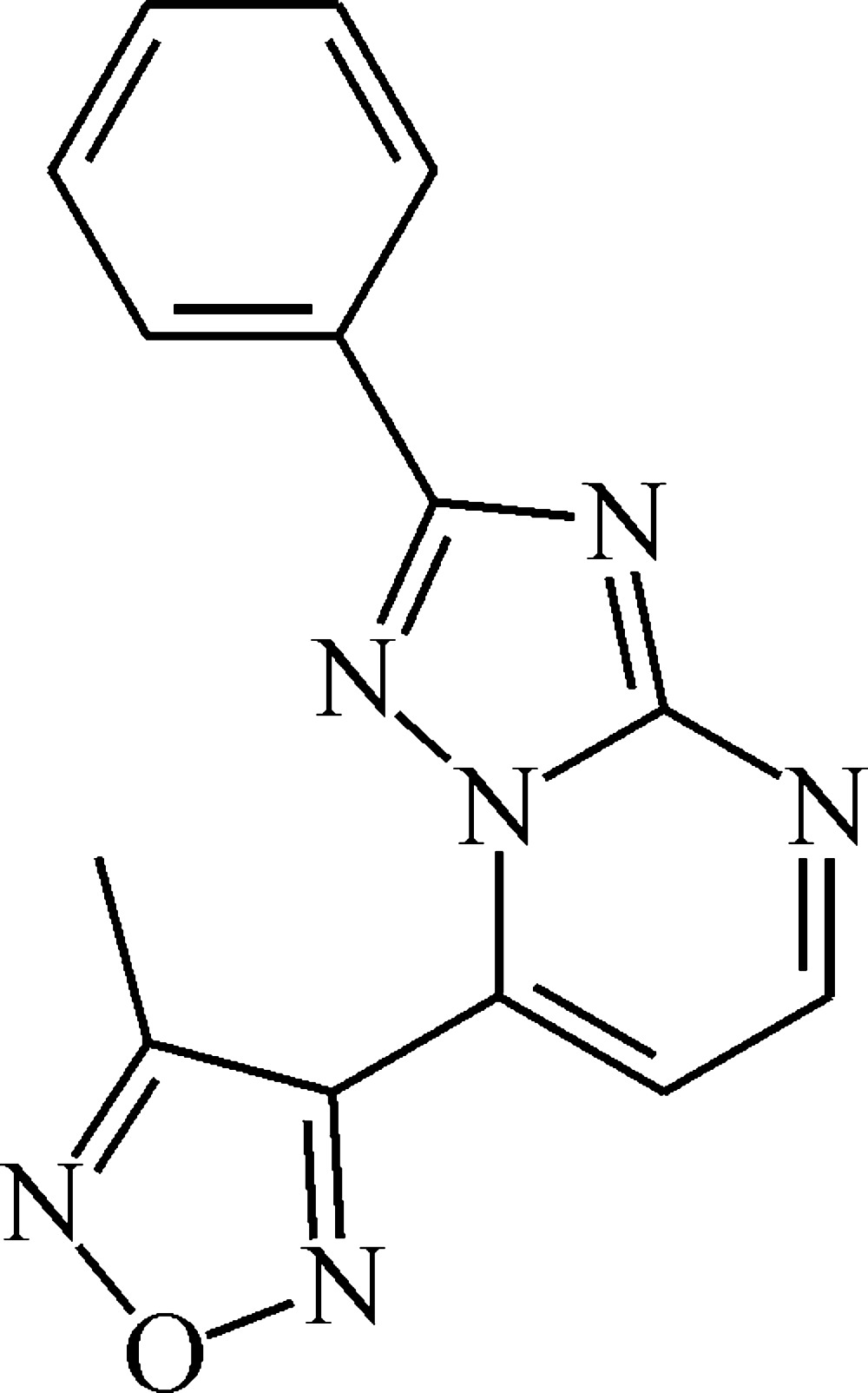


## Experimental

### 

#### Crystal data

C_14_H_10_N_6_O
*M* *_r_* = 278.28
Monoclinic, 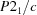

*a* = 11.1397 (6) Å
*b* = 15.6579 (8) Å
*c* = 7.3952 (4) Å
β = 101.332 (1)°
*V* = 1264.76 (12) Å^3^
*Z* = 4
Mo *K*α radiation
μ = 0.10 mm^−1^
*T* = 120 K
0.32 × 0.28 × 0.26 mm

#### Data collection

Bruker APEXII CCD diffractometer
16844 measured reflections
4041 independent reflections
3456 reflections with *I* > 2σ(*I*)
*R*_int_ = 0.030

#### Refinement

*R*[*F* ^2^ > 2σ(*F* ^2^)] = 0.042
*wR*(*F* ^2^) = 0.118
*S* = 1.02
4041 reflections
191 parameters
H-atom parameters constrained
Δρ_max_ = 0.42 e Å^−3^
Δρ_min_ = −0.28 e Å^−3^

### 

Data collection: *APEX2* (Bruker, 2009 ▶); cell refinement: *SAINT* (Bruker, 2009 ▶); data reduction: *SAINT*; program(s) used to solve structure: *SHELXTL* (Sheldrick, 2008 ▶); program(s) used to refine structure: *SHELXTL*; molecular graphics: *SHELXTL*; software used to prepare material for publication: *SHELXTL*, *publCIF* (Westrip, 2010 ▶) and *PLATON* (Spek, 2009 ▶).

## Supplementary Material

Crystal structure: contains datablock(s) I, New_Global_Publ_Block. DOI: 10.1107/S1600536813027700/cv5430sup1.cif

Structure factors: contains datablock(s) I. DOI: 10.1107/S1600536813027700/cv5430Isup2.hkl

Supplementary material file. DOI: 10.1107/S1600536813027700/cv5430Isup3.cml

Additional supplementary materials:  crystallographic information; 3D view; checkCIF report

## Figures and Tables

**Fig. 1.**
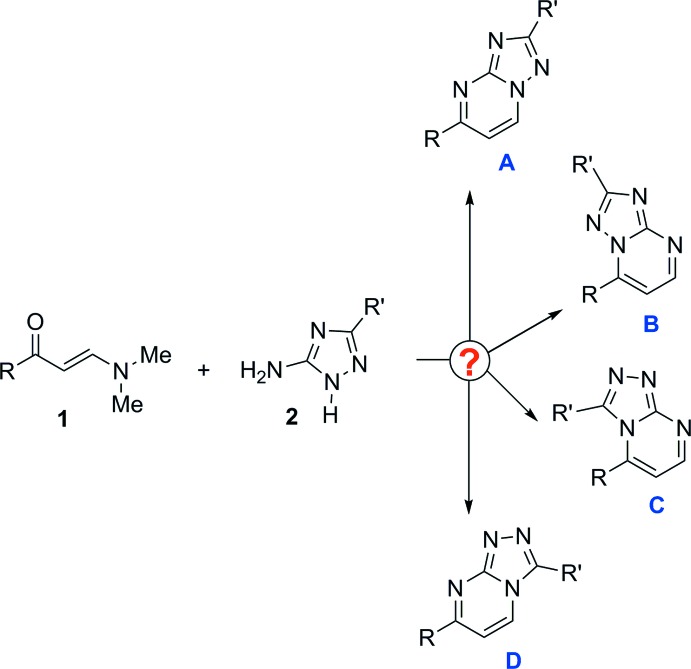
Possible types of triazolopyrimidines from the reaction of dimethylaminopropenone with 3-amino-1,2,4-triazoles.

**Fig. 2.**
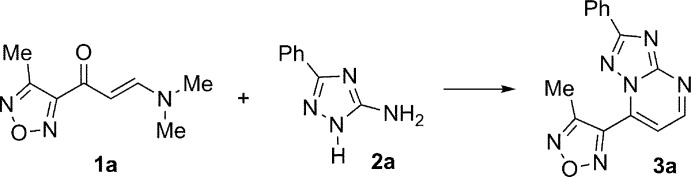
Synthesis of the title compound 3a.

**Fig. 3.**
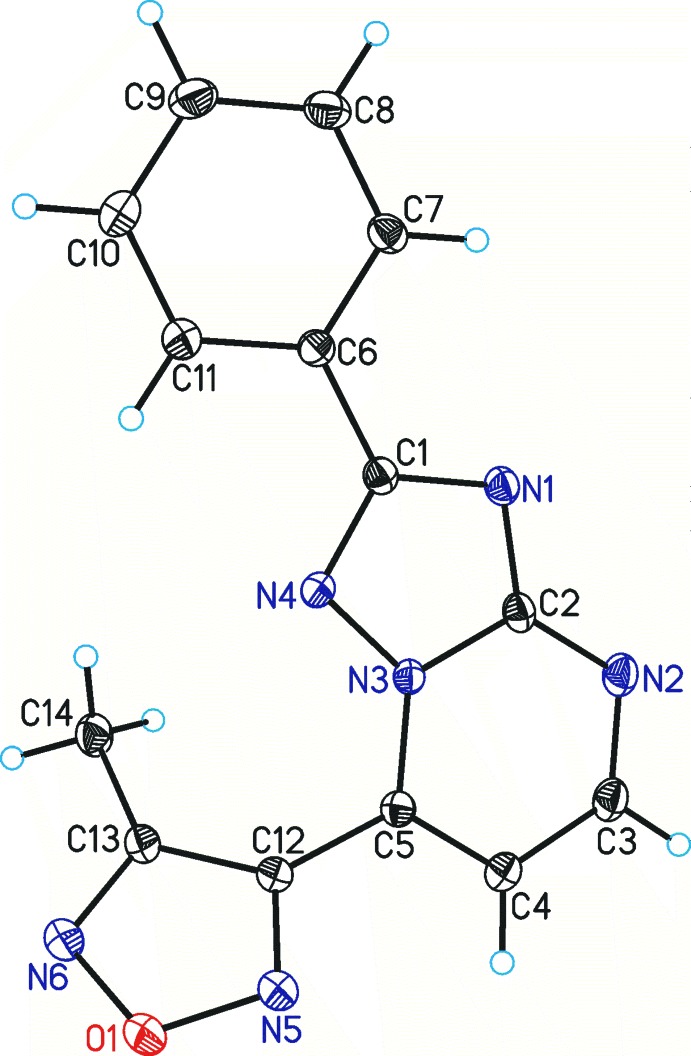
The molecular structure of the title compound with the atom numbering scheme. Displacement ellipsoids are drawn at the 50% probability level.

**Fig. 4.**
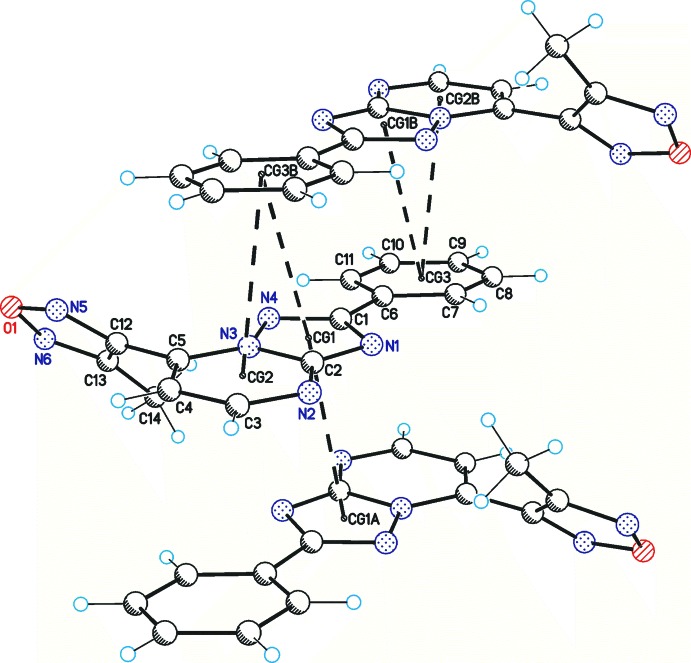
A portion of the crystal packing showing π–π stacking interactions by dashed lines. Cg1, Cg2 and Cg3 are centroids of C1/N1/C2/N3/N4, C2/N2/C3—C5/N3 and C6—C11 cycles, respectively. Symmetry codes: (A) -x + 2, -y, -z; (B) -x + 2, -y, -z + 1.

## supplementary crystallographic information

### 1. Comment

Dimethylaminopropenones have been widely employed as key building blocks in the synthesis of functionalized alkenes, aromatics and heterocycles, especially 1,2,4-triazolopyrimidines with potential medicinal application (Kulinich & Ischenko, 2009; Stanovnik & Svete, 2004). 1,2,4-Triazolopyrimidines can be obtained readily from the cyclocondensation of dimethylaminopropenones with 3-amino-1,2,4-triazoles. The cyclocondensation can, in principle, yield four regioisomers, *i.e.* 2-*R*'-5-*R*-[1,2,4]triazolo[1,5-*a*]pyrimidine (A), 2-*R*'-7-*R*-[1,2,4]triazolo[1,5-*a*]pyrimidine (B), 3-*R*'-5-*R*-[1,2,4]triazolo[4,3-*a*]pyrimidine (C) and 3-*R*'-7-*R*-[1,2,4]triazolo[4,3-*a*]pyrimidine (D) as depicted in Figure 1. Different structures have been assigned to products of the reaction in various studies: strucuture B (Behbehani & Ibrahim, 2012), C (Abdelhamid *et al.*, 2013) or D (Abdelhamid *et al.*, 2012). Thus recent literature indicated that the unambiguous identification of obtained regioisomer is problematic. However, up to now single-crystal X-ray analyses was not used to verify the assigned structures of the products obtained from cyclocondensation of dimethylaminopropenones with 3-amino-1,2,4-triazoles.

In the present study we found that cyclocondensation of 3-(dimethylamino)-1-(4-methylfurazan-3-yl)prop-2-en-1-one (1a) with 3-amino-5-phenyl-1,2,4-triazole (2a) in acetic acid resulted in formation of 3-methyl-4-(2-phenyl-[1,2,4]triazolo[1,5-*a*]pyrimidin-7-yl)-furazan (3a), *i.e.* regioisomer of type B (Figure 2).

According to X-ray data, nearly planar [1,2,4]triazolo[1,5-*a*]pyrimidine core form interplanar angle of 5.45 (4)° with the phenyl substituent. Methyl substituted furazan ring is rotated out of the triazolopyrimidine plane (torsional angle N3—C5—C12—C13 is equal to -136.53 (10)°) due to sterical repulsion between the methyl group and the triazolopyrimidine bicycle (Figure 3). In accordance with our earlier study on furazan derivatives (Sheremetev *et al.*, 2004, 2006, 2012, 2013; Suponitsky *et al.*, 2009*a*, 2009*b*) the O1—N6 and O1—N5 bond lengths of 1.3929 (12) and 1.3742 (12) Å, respectively, are normal. Bond lengths distribution in triazolopyrimidine core is similar to previously studied triazolopyrimidine derivatives (Lipkind *et al.*, 2011; Shikhaliev *et al.*, 2008; Lokaj *et al.*, 2006; Allen, 2002; Allen *et al.*, 1987).

In the crystal structure, along the crystallographic direction *c*, molecules form columns in which they are related by the center of symmetry and connected by alternating π–π stacking interactions (Figure 4). The stronger stacking interactions (interplanar distance is 3.306 (3) Å, the shortest contacts are C3···C6^i^ 3.3387 (13) Å; C1···C2^i^ 3.3580 (13) Å) connects molecules into dimers which are linked together by weaker stacking interactions (interplanar distance is 3.412 (3) Å, the shortest contacts are C5···C8^ii^ 3.3858 (14) Å; C1···C1^ii^ 3.370 (2) Å). Symmetry codes: (i) -*x* + 2, -*y*, -*z*; (ii) -*x* + 2, -*y*, -*z* + 1. Intercentroid distances are given in the Table 1.

### 2. Experimental

The crystals of the title compound suitable for X-ray analysis were grown by slow evaporation of tetraclormethane solution of the title compound.

All the reagents were of analytical grade, purchased from commercial sources, and used as received. Infrared spectra were determined in KBr pellets on a Perkin-Elmer Model 577 spectrometer. Mass-spectra were recorded on a Varian MAT-311 A instrument. The ^1^H and ^13^C NMR spectra were recorded at 300.13 and 75.47 MHz, respectively. The chemical shift values (*δ*, p.p.m.) are expressed relative to the chemical shift of the solvent-*d*. Melting points were determined on Gallenkamp melting point apparatus and are uncorrected.

3-(Dimethylamino)-1-(4-methylfurazan-3-yl)prop-2-en-1-one (1a). A mixture of 2-acetyl-3-methylfurazan (35 g, 0.277 mol) and dimethylformamide dimethylacetal (35 g, 0.294 mol) was refluxed in *o*-xylene (150 ml) for 3 h. The reaction mixture was then cooled and diluted with petroleum ether. The solid formed was collected by filtration and crystallized from ethanol. Yield 50.1 g (71%), mp 91–92 °C. IR (KBr), *ν*, cm^-1^: 1649, 1580, 1551, 1493, 1465, 1422, 1394, 1353, 1271, 1124, 1066, 1032, 1010, 981, 904, 882, 793, 776, 759. ^1^H MNR (CDCl_3_, *δ*, p.p.m.): 2.49 (s, 3H, CH_3_), 2.98 (s, 3H, CH_3_), 3.12 (s, 3H, CH_3_), 5.76 (d, 1H, CH), 7.74 (d, 1H, CH); ^13^C MNR (CDCl_3_, *δ*, p.p.m.): 9.2, 37.3, 45.2, 93.2, 151.4, 152.4, 154.4, 177.8. MS: m/*z* 181 (*M*^+^). Anal. Calcd. for C_8_H_11_N_3_O_2_ (181.19): C, 53.03; H, 6.12; N, 23.19. Found: C, 53.09; H, 6.07; N, 23.08.

3-Methyl-4-(2-phenyl-[1,2,4]triazolo[1,5-*a*]pyrimidin-7-yl)-furazan (3a). A mixture of compound 1a (0.36 g, 2 mmol), 3-amino-5-phenyl-1,2,4-triazole 2a (0.32 g, 2 mmol) and acetic acid (5 ml) was refluxed for 8 h. After cooling the product separated was collected by filtration and recrystallized from MeCN. Yield 0.36 g (65%), mp 209–210°C. IR (KBr), *ν*, cm^-1^: 1619, 1578, 1544, 1513, 1453, 1441, 1408, 1350, 1327, 1285, 1263, 1205, 1130, 1071, 964, 904, 844, 822, 773; ^1^H MNR (DMSO-*d*_6_, *δ*, p.p.m.): 2.62 (3*H*, CH_3_), 7.54 (3*H*, Ph), 7.78 (1*H*, CH), 8.20 (2*H*, Ph), 9.03 (1*H*, CH); ^13^C NMR (DMSO-*d*_6_, *δ*, p.p.m.): 10.01, 111.5, 127.5, 128.7, 129.5, 131.08, 134.5, 147.3, 151.2, 153.8, 156.4, 166.5. Anal. Calcd. for C_14_H_10_N_6_O (278.27): C, 60.43; H, 3.62; N, 30.20. Found: C, 60.56; H, 3.69; N, 30.07.

### 3. Refinement

All H atoms were geometrically positioned (C—H 095–0.98 Å), and refined as riding, with *U*_iso_(H) = 1.2–1.5 *U*_eq_(C).

### Crystal data

**Table:**

C_14_H_10_N_6_O	*F*(000) = 576
*M**_r_* = 278.28	*D*_x_ = 1.461 Mg m^−^^3^
Monoclinic, *P*2_1_/*c*	Melting point = 483–482 K
Hall symbol: -P 2ybc	Mo *K*α radiation, λ = 0.71073 Å
*a* = 11.1397 (6) Å	Cell parameters from 7123 reflections
*b* = 15.6579 (8) Å	θ = 2.3–31.0°
*c* = 7.3952 (4) Å	µ = 0.10 mm^−^^1^
β = 101.332 (1)°	*T* = 120 K
*V* = 1264.76 (12) Å^3^	Prizm, colourless
*Z* = 4	0.32 × 0.28 × 0.26 mm

### Data collection

**Table:**

Bruker APEXII CCD diffractometer	3456 reflections with *I* > 2σ(*I*)
Radiation source: sealed tube	*R*_int_ = 0.030
Graphite monochromator	θ_max_ = 31.0°, θ_min_ = 1.9°
φ and ω scans	*h* = −16→16
16844 measured reflections	*k* = −22→21
4041 independent reflections	*l* = −10→10

### Refinement

**Table:**

Refinement on *F*^2^	Primary atom site location: structure-invariant direct methods
Least-squares matrix: full	Secondary atom site location: difference Fourier map
*R*[*F*^2^ > 2σ(*F*^2^)] = 0.042	Hydrogen site location: inferred from neighbouring sites
*wR*(*F*^2^) = 0.118	H-atom parameters constrained
*S* = 1.02	*w* = 1/[σ^2^(*F*_o_^2^) + (0.0678*P*)^2^ + 0.3512*P*] where *P* = (*F*_o_^2^ + 2*F*_c_^2^)/3
4041 reflections	(Δ/σ)_max_ < 0.001
191 parameters	Δρ_max_ = 0.42 e Å^−^^3^
0 restraints	Δρ_min_ = −0.28 e Å^−^^3^

### Special details

**Table:**

Geometry. All e.s.d.'s (except the e.s.d. in the dihedral angle between two l.s. planes) are estimated using the full covariance matrix. The cell e.s.d.'s are taken into account individually in the estimation of e.s.d.'s in distances, angles and torsion angles; correlations between e.s.d.'s in cell parameters are only used when they are defined by crystal symmetry. An approximate (isotropic) treatment of cell e.s.d.'s is used for estimating e.s.d.'s involving l.s. planes.
Refinement. Refinement of *F*^2^ against ALL reflections. The weighted *R*-factor *wR* and goodness of fit *S* are based on *F*^2^, conventional *R*-factors *R* are based on *F*, with *F* set to zero for negative *F*^2^. The threshold expression of *F*^2^ > σ(*F*^2^) is used only for calculating *R*-factors(gt) *etc*. and is not relevant to the choice of reflections for refinement. *R*-factors based on *F*^2^ are statistically about twice as large as those based on *F*, and *R*- factors based on ALL data will be even larger.

### Fractional atomic coordinates and isotropic or equivalent isotropic displacement parameters (Å^2^)

**Table:**

	*x*	*y*	*z*	*U*_iso_*/*U*_eq_	
O1	0.56608 (7)	0.23558 (5)	0.09319 (14)	0.0282 (2)	
N1	1.00892 (7)	−0.06092 (5)	0.21751 (12)	0.01583 (17)	
N2	0.82706 (8)	−0.11973 (6)	0.02377 (12)	0.01834 (18)	
N3	0.84903 (7)	0.02462 (5)	0.13466 (11)	0.01300 (16)	
N4	0.93391 (7)	0.07409 (5)	0.24376 (11)	0.01399 (16)	
N5	0.58150 (8)	0.14848 (6)	0.09470 (14)	0.0227 (2)	
N6	0.67069 (8)	0.27675 (6)	0.06085 (15)	0.0239 (2)	
C1	1.02813 (8)	0.01935 (6)	0.28809 (13)	0.01377 (17)	
C2	0.89448 (9)	−0.05739 (6)	0.12102 (13)	0.01468 (18)	
C3	0.71487 (9)	−0.09738 (6)	−0.05783 (14)	0.01883 (19)	
H3A	0.6646	−0.1399	−0.1265	0.023*	
C4	0.66417 (9)	−0.01483 (6)	−0.05042 (13)	0.01698 (19)	
H4A	0.5829	−0.0031	−0.1129	0.020*	
C5	0.73399 (8)	0.04786 (6)	0.04821 (13)	0.01414 (17)	
C6	1.14510 (8)	0.04615 (6)	0.40276 (13)	0.01413 (17)	
C7	1.23825 (9)	−0.01412 (6)	0.45314 (14)	0.01694 (19)	
H7A	1.2241	−0.0721	0.4178	0.020*	
C8	1.35164 (9)	0.01047 (7)	0.55480 (15)	0.0205 (2)	
H8A	1.4147	−0.0307	0.5887	0.025*	
C9	1.37265 (10)	0.09538 (8)	0.60680 (16)	0.0247 (2)	
H9A	1.4504	0.1124	0.6748	0.030*	
C10	1.27942 (10)	0.15534 (7)	0.55890 (17)	0.0262 (2)	
H10A	1.2935	0.2132	0.5960	0.031*	
C11	1.16591 (9)	0.13117 (7)	0.45715 (15)	0.0202 (2)	
H11A	1.1027	0.1724	0.4247	0.024*	
C12	0.69232 (9)	0.13591 (6)	0.06503 (13)	0.01574 (18)	
C13	0.74940 (9)	0.21662 (6)	0.04368 (14)	0.01690 (19)	
C14	0.87253 (9)	0.23617 (7)	0.00342 (15)	0.0197 (2)	
H14A	0.8730	0.2945	−0.0444	0.030*	
H14B	0.8910	0.1958	−0.0887	0.030*	
H14C	0.9345	0.2310	0.1169	0.030*	

### Atomic displacement parameters (Å^2^)

**Table:**

	*U*^11^	*U*^22^	*U*^33^	*U*^12^	*U*^13^	*U*^23^
O1	0.0170 (4)	0.0169 (4)	0.0508 (5)	0.0032 (3)	0.0073 (3)	−0.0001 (3)
N1	0.0163 (4)	0.0127 (4)	0.0189 (4)	0.0008 (3)	0.0044 (3)	−0.0004 (3)
N2	0.0204 (4)	0.0139 (4)	0.0210 (4)	−0.0017 (3)	0.0048 (3)	−0.0031 (3)
N3	0.0129 (3)	0.0107 (3)	0.0156 (3)	−0.0004 (3)	0.0032 (3)	−0.0006 (3)
N4	0.0126 (3)	0.0128 (3)	0.0164 (4)	−0.0012 (3)	0.0023 (3)	−0.0010 (3)
N5	0.0162 (4)	0.0155 (4)	0.0359 (5)	0.0014 (3)	0.0036 (4)	0.0000 (3)
N6	0.0185 (4)	0.0154 (4)	0.0372 (5)	0.0013 (3)	0.0040 (4)	0.0016 (4)
C1	0.0139 (4)	0.0129 (4)	0.0153 (4)	0.0001 (3)	0.0050 (3)	0.0010 (3)
C2	0.0169 (4)	0.0114 (4)	0.0168 (4)	0.0007 (3)	0.0058 (3)	−0.0004 (3)
C3	0.0205 (4)	0.0147 (4)	0.0211 (4)	−0.0034 (3)	0.0038 (4)	−0.0034 (3)
C4	0.0164 (4)	0.0158 (4)	0.0182 (4)	−0.0022 (3)	0.0022 (3)	−0.0011 (3)
C5	0.0138 (4)	0.0133 (4)	0.0154 (4)	−0.0002 (3)	0.0031 (3)	0.0011 (3)
C6	0.0126 (4)	0.0144 (4)	0.0160 (4)	0.0000 (3)	0.0045 (3)	0.0017 (3)
C7	0.0157 (4)	0.0165 (4)	0.0192 (4)	0.0016 (3)	0.0049 (3)	0.0037 (3)
C8	0.0145 (4)	0.0233 (5)	0.0233 (5)	0.0013 (4)	0.0026 (4)	0.0067 (4)
C9	0.0168 (4)	0.0266 (5)	0.0283 (5)	−0.0043 (4)	−0.0016 (4)	0.0042 (4)
C10	0.0222 (5)	0.0192 (5)	0.0340 (6)	−0.0043 (4)	−0.0018 (4)	−0.0021 (4)
C11	0.0169 (4)	0.0159 (4)	0.0269 (5)	0.0005 (3)	0.0020 (4)	−0.0009 (4)
C12	0.0140 (4)	0.0136 (4)	0.0184 (4)	0.0003 (3)	0.0004 (3)	0.0006 (3)
C13	0.0172 (4)	0.0132 (4)	0.0193 (4)	0.0003 (3)	0.0012 (3)	0.0011 (3)
C14	0.0203 (4)	0.0154 (4)	0.0243 (5)	−0.0012 (3)	0.0068 (4)	0.0030 (4)

### Geometric parameters (Å, º)

**Table:**

O1—N5	1.3742 (12)	C5—C12	1.4678 (13)
O1—N6	1.3929 (12)	C6—C11	1.3968 (14)
N1—C2	1.3342 (12)	C6—C7	1.3979 (13)
N1—C1	1.3616 (12)	C7—C8	1.3914 (14)
N2—C3	1.3236 (13)	C7—H7A	0.9500
N2—C2	1.3497 (12)	C8—C9	1.3910 (16)
N3—N4	1.3583 (11)	C8—H8A	0.9500
N3—C5	1.3643 (12)	C9—C10	1.3931 (16)
N3—C2	1.3912 (12)	C9—H9A	0.9500
N4—C1	1.3449 (12)	C10—C11	1.3909 (14)
N5—C12	1.3106 (13)	C10—H10A	0.9500
N6—C13	1.3095 (13)	C11—H11A	0.9500
C1—C6	1.4693 (13)	C12—C13	1.4373 (13)
C3—C4	1.4160 (14)	C13—C14	1.4914 (14)
C3—H3A	0.9500	C14—H14A	0.9800
C4—C5	1.3702 (13)	C14—H14B	0.9800
C4—H4A	0.9500	C14—H14C	0.9800
			
Cg1···Cg1^i^	3.456 (2)	Cg1···Cg3^ii^	3.591 (2)
Cg2···Cg3^ii^	3.540 (2)		
			
N5—O1—N6	110.69 (8)	C8—C7—C6	120.31 (9)
C2—N1—C1	103.20 (8)	C8—C7—H7A	119.8
C3—N2—C2	115.36 (9)	C6—C7—H7A	119.8
N4—N3—C5	127.33 (8)	C7—C8—C9	119.96 (9)
N4—N3—C2	110.41 (8)	C7—C8—H8A	120.0
C5—N3—C2	122.26 (8)	C9—C8—H8A	120.0
C1—N4—N3	101.55 (7)	C10—C9—C8	119.82 (10)
C12—N5—O1	105.55 (8)	C10—C9—H9A	120.1
C13—N6—O1	106.41 (8)	C8—C9—H9A	120.1
N4—C1—N1	116.01 (8)	C11—C10—C9	120.48 (10)
N4—C1—C6	121.32 (8)	C11—C10—H10A	119.8
N1—C1—C6	122.66 (8)	C9—C10—H10A	119.8
N1—C2—N2	128.91 (9)	C10—C11—C6	119.80 (10)
N1—C2—N3	108.82 (8)	C10—C11—H11A	120.1
N2—C2—N3	122.27 (9)	C6—C11—H11A	120.1
N2—C3—C4	124.87 (9)	N5—C12—C13	109.75 (9)
N2—C3—H3A	117.6	N5—C12—C5	118.64 (9)
C4—C3—H3A	117.6	C13—C12—C5	131.49 (9)
C5—C4—C3	119.05 (9)	N6—C13—C12	107.61 (9)
C5—C4—H4A	120.5	N6—C13—C14	122.10 (9)
C3—C4—H4A	120.5	C12—C13—C14	130.27 (9)
N3—C5—C4	116.17 (9)	C13—C14—H14A	109.5
N3—C5—C12	119.63 (8)	C13—C14—H14B	109.5
C4—C5—C12	124.20 (9)	H14A—C14—H14B	109.5
C11—C6—C7	119.61 (9)	C13—C14—H14C	109.5
C11—C6—C1	121.11 (9)	H14A—C14—H14C	109.5
C7—C6—C1	119.25 (9)	H14B—C14—H14C	109.5
			
C5—N3—N4—C1	−179.38 (9)	N4—C1—C6—C11	−5.31 (14)
C2—N3—N4—C1	1.14 (9)	N1—C1—C6—C11	173.36 (9)
N6—O1—N5—C12	0.26 (12)	N4—C1—C6—C7	176.72 (9)
N5—O1—N6—C13	−0.33 (12)	N1—C1—C6—C7	−4.61 (13)
N3—N4—C1—N1	−0.77 (10)	C11—C6—C7—C8	−0.83 (14)
N3—N4—C1—C6	177.98 (8)	C1—C6—C7—C8	177.17 (9)
C2—N1—C1—N4	0.08 (11)	C6—C7—C8—C9	0.02 (15)
C2—N1—C1—C6	−178.65 (8)	C7—C8—C9—C10	0.85 (17)
C1—N1—C2—N2	−178.95 (10)	C8—C9—C10—C11	−0.91 (18)
C1—N1—C2—N3	0.65 (10)	C9—C10—C11—C6	0.09 (17)
C3—N2—C2—N1	179.64 (9)	C7—C6—C11—C10	0.77 (15)
C3—N2—C2—N3	0.09 (14)	C1—C6—C11—C10	−177.19 (10)
N4—N3—C2—N1	−1.19 (10)	O1—N5—C12—C13	−0.09 (12)
C5—N3—C2—N1	179.29 (8)	O1—N5—C12—C5	−176.58 (9)
N4—N3—C2—N2	178.44 (8)	N3—C5—C12—N5	−136.53 (10)
C5—N3—C2—N2	−1.08 (14)	C4—C5—C12—N5	43.38 (14)
C2—N2—C3—C4	0.60 (15)	N3—C5—C12—C13	47.89 (15)
N2—C3—C4—C5	−0.33 (15)	C4—C5—C12—C13	−132.19 (11)
N4—N3—C5—C4	−178.14 (9)	O1—N6—C13—C12	0.26 (11)
C2—N3—C5—C4	1.30 (13)	O1—N6—C13—C14	178.61 (9)
N4—N3—C5—C12	1.79 (14)	N5—C12—C13—N6	−0.11 (12)
C2—N3—C5—C12	−178.78 (8)	C5—C12—C13—N6	175.77 (10)
C3—C4—C5—N3	−0.63 (13)	N5—C12—C13—C14	−178.28 (10)
C3—C4—C5—C12	179.46 (9)	C5—C12—C13—C14	−2.40 (18)

Symmetry codes: (i) −*x*+2, −*y*, −*z*; (ii) −*x*+2, −*y*, −*z*+1.
